# Associations between the gut microbiome and fatigue in cancer patients

**DOI:** 10.1038/s41598-021-84783-9

**Published:** 2021-03-12

**Authors:** Joud Hajjar, Tito Mendoza, Liangliang Zhang, Siqing Fu, Sarina A. Piha-Paul, David S. Hong, Filip Janku, Daniel D. Karp, Alexej Ballhausen, Jing Gong, Abdulrazzak Zarifa, Christine B. Peterson, Funda Meric-Bernstam, Robert Jenq, Aung Naing

**Affiliations:** 1 Department of Pediatrics, Baylor College of Medicine, Houston, TX USA; 2 The William T. Shearer Center for Human Immunobiology, Texas Children’s Hospital, Houston, TX USA; 3 Department of Symptom Research, The University of Texas MD Anderson Cancer Center, Houston, TX USA; 4 Department of Biostatistics, The University of Texas MD Anderson Cancer Center, Houston, TX USA; 5 Department of Investigational Cancer Therapeutics, Unit 0455, The University of Texas MD Anderson Cancer Center, 1515 Holcombe Blvd, Houston, TX 77030 USA; 6 Department of Genomic Medicine, The University of Texas MD Anderson Cancer Center, Houston, TX USA

**Keywords:** Cancer, Cancer

## Abstract

Fatigue is the most prevalent symptom of cancer and its treatments. Changes in the intestinal microbiome have been identified in chronic fatigue syndrome and other neuropsychiatric disorders, and cancer patients. However, the association between intestinal microbiome and fatigue in patients with advanced cancers has not been evaluated. Understanding the connection between intestinal microbiome and fatigue will provide interventional and therapeutic opportunities to manipulate the microbiome to improve fatigue and other patients’ reported outcomes. In this project, we aimed to identify associations between microbiome composition and fatigue in advanced cancer patients. In this cross-sectional observational study at a tertiary cancer care center, we included 88 patients with advanced, metastatic, unresectable cancers who were in a washout period from chemotherapy. We measured fatigue using the MD Anderson Symptom Inventory—Immunotherapy fatigue score, and used 16srRNA to analyze intestinal microbiome. Using correlation analysis we found that *Eubacterium hallii* was negatively associated with fatigue severity scores (r = − 0.30, p = 0.005), whereas *Cosenzaea* was positively associated with fatigue scores (r = 0.33, p = 0.0002). We identified microbial species that exhibit distinct composition between high-fatigued and low-fatigued cancer patients. Further studies are warranted to investigate whether modulating the microbiome reduces cancer patients’ fatigue severity and improves their quality of life.

## Introduction

Fatigue is one of the most common symptoms of cancer, and its treatment. Having fatigue has been associated with worse cancer outcomes^1–6^. The interaction between microbial communities and the host (directly via microbial metabolites or indirectly via the immune system) supplies the central nervous system with real-time information about the environment^7^ and connects the emotional and cognitive centers of the brain with peripheral intestinal functions^8^.

Multiple studies have shown that the intestinal microbiome of cancer patients is different from that of patients without cancer. In addition, gut microbial dysbiosis prior to radiation therapy may predict fatigue in cancer patients^9^, and chemoradiotherapy can influence colorectal cancer patients’ intestinal microbiome composition and contribute to fatigue^10^.

Fatigue is the most prevalent and pervasive symptom caused by cancer and its treatments. Although commendable efforts have been made to diagnose cancer-related fatigue, the precise cause of fatigue in cancer patients remain elusive and treatment is moderately effective, at best^1,4,11–14^. Given recent research showing that among these patients, fatigue reduces quality of life and increases mortality^2,5,14^, understanding the mechanism(s) of cancer-related fatigue are imperative to improve both patient experience and outcomes.

Therefore, we conducted a retrospective study of fatigue in patients with advanced cancer that lead to identification of associations between microbiota parameters and fatigue severity in cancer patients. Identifying intestinal microbiome features that are associated with fatigue in cancer patients may improve our understanding of the mechanisms underlying fatigue and lead to the development of therapeutic approaches targeting the microbiome to prevent or reduce fatigue in these patients.

## Methods

### Patients

Patients were recruited from MD Anderson’s Department of Investigational Cancer Therapeutics between February 28, 2017 and January 4, 2018. Eligible patients were at least 18 years old, able to speak English, and had pathological diagnoses of advanced, metastatic, unresectable cancers. Patients had to be off antibiotics for at least 30 days before enrollment. Patients were excluded if clinical research staff believed that they did not understand the intent of the study or could not complete the symptom assessment measure. Patients’ clinical and demographic data were obtained by review of their electronic medical records including cancer diagnosis, prior use of antibiotics, probiotics, medications (including antidepressants), neuropsychiatric disorders, gastrointestinal disease, and prior anti-cancer treatment. The patients underwent protocol-required wash out period before enrollment, which is typically 4 weeks or 5 half-lives of the prior therapy.

### Fatigue measurement

Fatigue was assessed using the MD Anderson Symptom Inventory (MDASI)-immunotherapy module (MDASI-Immunotherapy), which has been validated for the assessment of 20 symptoms, including seven immunotherapy-specific items and six interference items^15^. Patients completed the module before they started immunotherapy and rated their symptoms and interference on a scale of 0–10 (0 = no symptom or no interference, 10 = worst symptom imaginable or complete interference). Using responses to the fatigue item, we categorized patients as having low fatigue (scores of 0–4) or high fatigue (scores of 5–10). The MDASI-Immunotherapy for early phase trials (EPT) has been shown to be valid and reliable^15^. In addition, it has been shown that the fatigue experience and its impact are reported along a single dimension and can be expressed as a single number^16^.

### Stool collection and microbiome analysis

Stool samples were collected at baseline using the OMNIgene-GUT kit (DNA Genotek, Inc.) and then stored at − 80 °C. Genomic bacterial DNA extraction methods were optimized to maximize the yield of bacterial DNA while keeping background amplification to a minimum. DNA extraction and bacterial 16S rRNA sequencing were performed as described previously^17^. Briefly, bacterial genomic DNA was extracted using the QIAamp DNA FFPE Tissue Kit (Qiagen). The 16S rDNA V4 region was amplified by polymerase chain reaction and sequenced using the MiSeq platform (Illumina). Between 4350 and 30,843 sequences were obtained for each sample (average 12,517). We have rarefied the OTU counts with a minimum of 4000 sequences across samples when calculating the Alpha Diversity, since this metric is sensitive to differences in sequencing depth. We have used the full unrarefied data for all the other downstream analyses, to avoid losing important information in the full microbiome data. To account for differences in read depth, we obtained the OTU relative abundances by scaling the OTU counts by their total counts in each sample.

As described in Wang, Y et al.^18^, we adopted the same pipeline to implement the microbiome analysis. VSEARCH was used for analyzing nucleotide sequences^19^. Paired-end reads were merged, de-replicated, and sorted by length and size. Sequences were then quality-controlled, error-corrected and chimera-filtered using the UNOISE algorithm to generate a preliminary list of OTUs. Both OTUs and presumed chimeras were assigned taxonomy in QIIME^20^ using the Mothur method^21^ with the Silva database version 128^22^. In addition, sequences rejected by the UNOISE algorithm that matched a database entry with a perfect score were restored to generate the final list of OTUs. We utilize UNOISE3 to computationally correct for PCR errors, which come in the form of mutations and chimeras. However, UNOISE3 does not always perform perfectly, especially with chimera calling. To compensate for this, our pipeline includes a step that maps rejected sequences to the curated 16S database Silva, and those rare rejected sequences that are found have a 100% identical match in the dataset are no longer classified as noise and are added back to the other sequences.

The generated OTU table, the clustered phylogenetic tree and the assigned taxonomy were then loaded into R 3.6.1 for additional quantitative analysis. The individual OTU counts were normalized by the total OTU counts in each sample, thus the scaled OTU abundance vector sums up to one. Alpha diversity scores and UniFrac distance^23^ between samples were determined using Phyloseq^24^ and Vegan^25^ R packages. All the other analyses, including principle coordinate analysis (PCoA), testing procedures and visualization results, were performed and produced in R.

### Statistical analysis

We used statistical tests to compare demographic and clinical characteristics of the low- and high-fatigue patients. Categorical variables were evaluated using a chi-square test or a proportion test. The Mann–Whitney U test was used to compare the distributions of continuous variables between the low- and high-fatigue groups. Continuous variables were associated using Spearman correlation analyses^26^. All tests were two-sided. *P*-values less than 0.05 were considered statistically significant.

Alpha and beta diversity were compared between groups using non-parametric tests and PCoA was used for data visualization. Unlike principal component analysis, which uses Euclidean distances, PCoA allows a choice of distance metrics, including distances designed for microbiome data such as the weighted Unifrac metric. We used three alpha-diversity metrics: the number of species observed in the sample, the Shannon index, and the Inverse Simpson index. Differential analysis of alpha diversity measures differences in the richness and evenness of microbial composition between groups of interest. Beta diversity measures the structural change in microbial compositions from one sample to another. We performed the permutational analysis of variance (PERMANOVA) to test for differences in beta diversities between groups of interest.

We categorized patients as having low fatigue (i.e., having MDASI-Immunotherapy fatigue scores of 0–4) or high fatigue (i.e., having scores of 5–10). Because microbial data are high-dimensional and heterogeneous, we need to robustly identify features that were significantly different between these two groups^19,27^. Therefore, we used a systematic way to visualize and compare microbial data between the low- and high-fatigue groups. The “progressive permutation” method progressively permutes the grouping factor labels of the microbiome and performs multiple Mann–Whitney U tests in each scenario. The signal strengths of the top hits from the original data are compared with their performance in the permutated data; if the top hits are true positives identified from the data, a decreasing trend will emerge. Using this method, we not only evaluated the overall associations between the microbiome features and fatigue severity but also identified individual features that were robustly different between the two groups.

The fragility index is a measure of the robustness of the results of a clinical trial^28,29^. We used a similar concept in our progressive permutation analysis to evaluate the robustness of each significant taxon. The fragility index of a variable is the minimum number of permutation steps that would change the variable’s significance to non-significance. In the present study, the larger the fragility index was, the more stable the identified taxa were. Therefore, we ranked the importance of the taxa by their fragility indices. All the statistical analyses, including alpha and beta diversity analyses, and Mann − Whitney U tests, were performed in R version 3.6.1.

### Ethics approval and consent to participate

The protocol was approved by the Institutional Review Board at The University of Texas MD Anderson Cancer Center. The study was conducted in accordance with the Declaration of Helsinki and the International Conference on Harmonization Good Clinical Practice guidelines. All the study participants provided written informed consent before enrollment.

## Results

The characteristics of the 88 patients (45 women and 43 men) enrolled in the study are given in Table 1. The patients’ median age was 58.5 years. Most of the patients were white (n = 73) . The most frequent diagnoses were colon cancer, ovarian cancer, cervical cancer, and non-small cell lung cancer (Figure S1 in the supplementary).

**Table 1:** Patient characteristics.

Characteristic	All patients (n = 88)	Low-fatigue patients (n = 58)	High-fatigue patients (n = 30)	P-value
Median age (range), years	58.5 (22–79)	58.5 (23–78)	58 (22–79)	0.95
**Sex (%)**				0.2
Female	45 (51)	33 (57)	12 (40)	2.00E−05
Male	43 (49)	25 (43)	18 (60)	0.2
**Race (%)**				0.01
Asian	3 (3)	3 (5)	0	0.20
Black	7 (8)	2 (3)	5 (17)	0.03
White	73 (83)	52 (90)	21 (70)	0.02
Other	5 (6)	1 (2)	4 (13)	0.02
Median MDASI score (range)	3 (0–10)	2.5 (0–4)	6 (5–10)	< 0.001
**ECOG score (%)**				0.86
0	8 (9)	6 (10)	2 (7)	0.13
1	80 (91)	52 (90)	28 (93)	3.00E−04
Median hemoglobin level (range), gm/dL	11.9 (9–15.6)	11.9 (9.0–15.6)	11.9 (9.9–14.3)	0.98
Median albumin level (range), gm/dL	4.1 (3–4.9)	4.1 (3–4.9)	4.05 (3.2–4.7)	0.55
Median creatinine level (range), mg/dL	0.87 (0.53–1.89)	0.82 (0.53–1.89)	0.92 (0.58–1.53)	0.08

### MDASI-immunotherapy fatigue scores

Of the 88 patients, 58 (66%) were categorized as having low fatigue (9 had no fatigue and 49 had mild fatigue) and 30 (34%) were categorized as having high fatigue (18 had moderate fatigue and 12 had severe fatigue). The median MDASI-Immunotherapy fatigue score was 3. The mean MDASI-Immunotherapy fatigue score was 3.6 (standard deviation [SD] = 2.3). The median MDASI-Immunotherapy fatigue score was 6 for patients in the high fatigue group and was 2.5 for those in the low fatigue group.

### Alpha and beta diversity comparison

**Figure 1:**
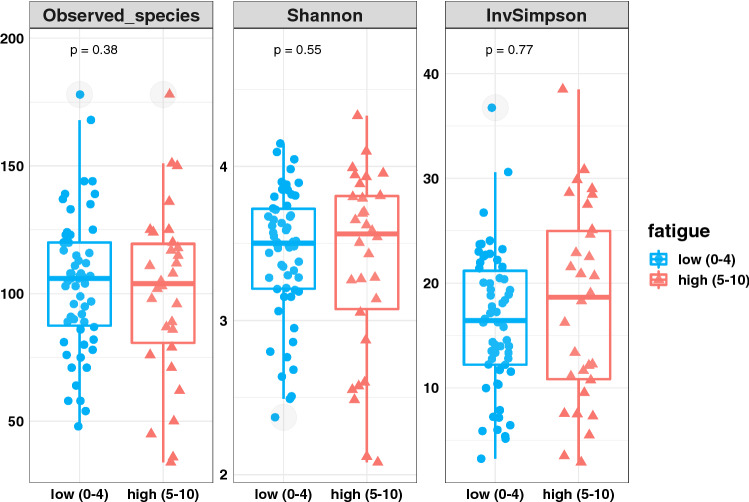
Box plots showing differences in alpha diversity between low- and high-fatigue patients. Each blue dot represents one patient in the low-fatigue group; each red triangle represents one patient in the high-fatigue group. The box-and-whisker plot describes the distribution of data points, including minimum, maximum, median, first quantile and third quantile. Grey shaded circles denote outliers.

**Figure 2:**
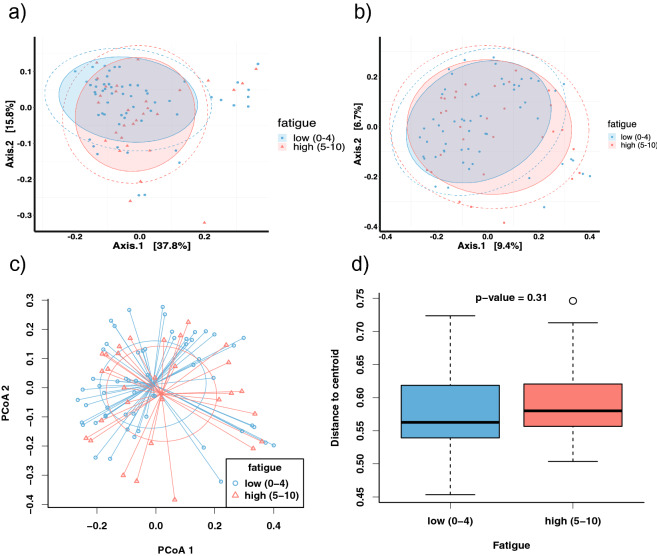
Results of the principal coordinate analysis (PCoA) showing differences in beta diversity between low-and high-fatigue patients. (**a**) PCoA plots using weighted-UniFrac metric. The x-axis explains 37.8% variation of the data. The y-axis explains 15.8% variation of the data. Each blue dot represents the PCoA score of one patient in the low-fatigue group; Each red triangle represents the PCoA score of one patient in the high-fatigue group. (**b**) PCoA plots using Bray–Curtis metric. The x-axis explains 9.4% variation of the data. The y-axis explains 6.7% variation of the data. The shaded ellipse denote the 80% confidence region of all the points. The dashed ellipse denote the 90% confidence region of all the points. (**c**) PCoA plot with individual distances to the centroid shows the inter-individual variations. Each blue dot represents the PCoA score of one patient in the low-fatigue group; Each red triangle represents the PCoA score of one patient in the high-fatigue group. (**d**) Boxplot of individual-centroid distances. The p-value is given by Tukey test. The box with blue color describes the distance distribution in the low fatigue group; The box with red color describes the distance distribution in the high fatigue group. (**c**,**d**) showed results from Bray–Curtis metric.

The alpha diversity analysis revealed no significant differences in microbiome diversity between low- and high-fatigue patients (observed species, p = 0.2; Shannon index, p = 0.57; Inverse Simpson index p = 0.78; Fig. 1). The beta diversity analysis revealed no significant differences in microbial composition between low- and high-fatigue patients. Because PERMANOVA generated the unsignificant results for both the weighted-UniFrac metric (r^2^ = 0.01, p = 0.45, Fig. 2a)) and the Bray–Curtis metric (r^2^ = 0.01, p = 0.40, Fig. 2b)). To compare the inter-individual variations between groups, we have added the PCoA plot with individual distances to the centroid (Fig. 2c)) and the boxplot of inter-individual distances (Fig. 2d)). Figure c,d showed results from Bray–Curtis metric. The Tukey test concluded a nonsignificant result (p = 0.31). We also included the two types of plots for weighted-UniFrac metric in the supplementary (Figure S3). The Tukey test also concluded a nonsignificant result (p = 0.51).

### Differences in the relative abundance of microbial taxa

Microbial features were agglomerated across the taxonomic levels phylum, class, order, family, genus, and species. The taxonomic compositions in low- and high-fatigue groups at phylum-class-order-family-genus levels are shown in Figure S2 (in the supplementary). Mann–Whitney U tests identified 15 of these features to be significantly differential between low- and high-fatigue groups (p < 0.05; Figure S4). Among the 24 features, a subgroup of 12 features (labelled in Figure S4) were identified to be robust findings based on the progressive permutation analysis. The trending curve of P-Values, the fragility indices and the effect sizes are shown in Figure S5 in the supplementary.

**Figure 3:**
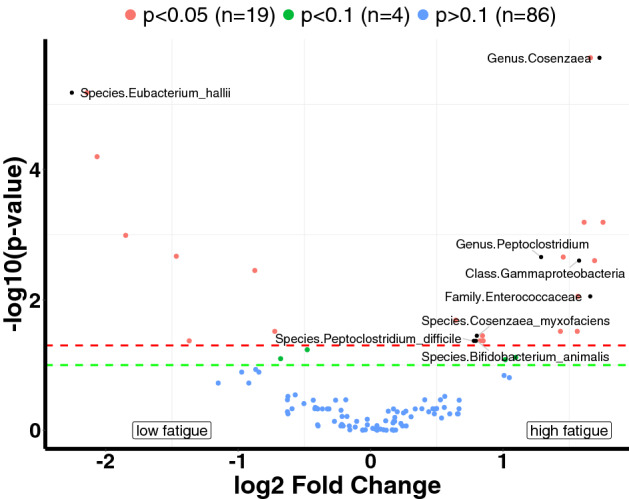
Volcano plot showing that, of 109 taxonomic features, 19 were significantly differential between low- and high-fatigue groups (p < 0.05; DESeq test with Benjamini–Hochberg procedure to adjust the p-values). Eight features identified by DESeq agree with the ones identified by our method. They were annotated with text names in the plot. The blue dot represents the features whose p-values are greater than 0.1; the green dot represents the features whose p-values are less than 0.1 and greater than 0.05; the red dot represents the features whose p-value are less than 0.05. A positive effect size indicates that the microbial feature is more abundant in high-fatigue group.

To verify the results, we also applied the DESeq method^30^, with the Benjamini–Hochberg procedure used to adjust the p-values^31^. We presented the adjusted p-values in the volcano plot (Fig. 3). Eight features identified by DESeq agree with the ones identified by our Wilcoxon method. We also applied LEfSe and presented the results in the barplot (Figure S6 in the supplementary). LEfSe uses both p-values and LDA scores to determine hits; however, it does not adjust the p-values for multiplicity. Using the default threshold of 2 on the LDA scores^32^, LEfSe selected 21 features in total. Eleven features identified by LEfSe agree with the ones identified by our method. We have also performed indicator species analysis using the labdsv package in R. We have plotted the selected variables and their − log10 p-values, as shown in the supplementary (Figure S6 in the supplementary). We still found five same features that are identified by our method.

**Figure 4:**
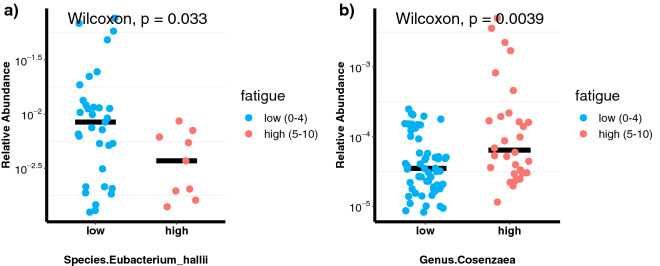
(**a**) Dot plots showing differences in relative abundance in *Eubacterium hallii* abundance between the high- and low-fatigue groups. Each blue dot represents one patient in the low-fatigue group; each red dot represents one patient in the high-fatigue group. The black bar denotes the median. (**b**) Dot plots showing differences in relative abundance in *Cosenzaea* abundance between the high- and low-fatigue groups. Each blue dot represents one patient in the low-fatigue group; each red dot represents one patient in the high-fatigue group. The black bar denotes the median. In both figures, we performed log10 transformation of the data so that the data points are evenly distributed. The numbers on the y-axis denotes the original quantity of relative abundance, which are just the scientific format of the abundance proportions.

We studied and plotted the distribution across samples of these 12 features one-by-one in Fig. 4 and Figure S11 in the supplementary. *Eubacterium hallii and Cosenzaea* were most differential between the high- and low-fatigue groups (Fig. 4). Both of them are also identified by all the above testing procedures. Compared with the low-fatigue group, the high-fatigue group had a significantly lower abundance of *Eubacterium hallii* (p = 0.005; Fig. 4a) but a significantly higher abundance of *Cosenzaea* (p = 0.0039; Fig. 4b). The abundances of *Eubacterium hallii* and *Cosenzaea* in each patient sample grouped by fatigue severity are shown in Figures S4A and S4B in the supplementary. Correlation analysis revealed that *Eubacterium hallii* was negatively associated with fatigue severity score (r = − 0.31, p = 0.0026), whereas *Cosenzaea* was positively associated with fatigue severity score (r = 0.26, p = 0.014). As the correlation analysis is mainly meant for visualization and exploration instead of inference, the p-values in the correlation analysis are not adjusted to correct for multiple testing.

We performed the Random Forest method to check the classification accuracy using the 12 selected features. We randomly split the data into 70% training set and 30% testing set. We repeated the training and testing procedure for 50 times. We have added the plot of the importance of these selected features in Figure S9 in the supplementary. It shows that the taxonomic feature “Species.Clostridium_dakarense” has the highest importance. We performed SpiecEasi (SParse InversE Covariance Estimation for Ecological Association Inference) to infer the network structures. Compared with CCREPE and SparCC, which are focused on estimation of unconditional correlation, SpiecEasi reliably estimates a sparse inverse covariance which can model the conditional independence between microbial features^33^. We have added the plot of the network at the genus level (178 features) in Figure S8 in the supplementary. When comparing the two networks, the low fatigue group network includes 285 edges, while the high fatigue group network includes 345 edges. This indicates that there are more co-occurring genera found in the high-fatigue group.

**Figure 5:**
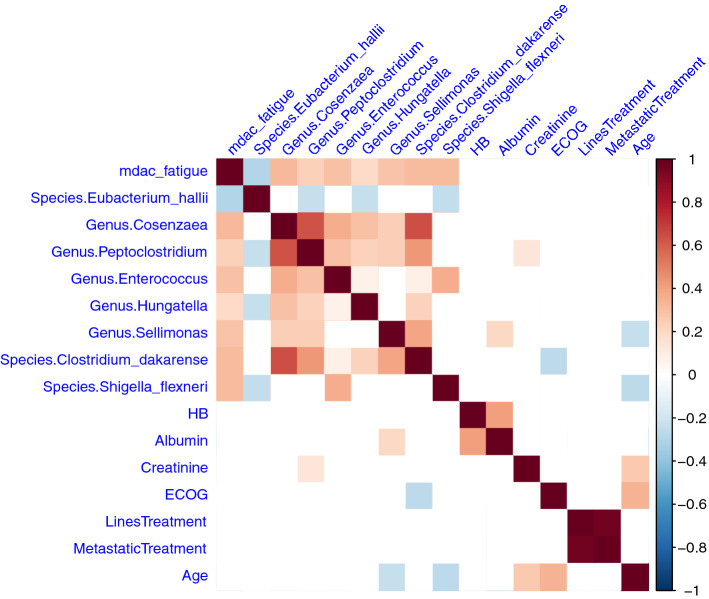
Results of the Spearman correlation analysis showing relationships among fatigue, selected microbiome species, and clinical measurements. The blue color indicates negative correlation while the red color indicates positive correlation. The depth of the color describes the strength of correlation. The absence of a color indicates a lack of no correlation (p-value > 0.05).

We performed Spearman correlation analysis to show the correlations between microbial abundances and clinical factors. The taxa that were resolved till genus and species levels were shown in the correlation plot (Fig. 5). The results revealed that microbiome features were not strongly correlated with clinical factors that are potentially associated with fatigue severity, including hemoglobin level, albumin level, creatinine level, Eastern Cooperative Oncology Group performance status score, number of lines of treatment, metastatic status, and age (Fig. 5). We have provided scatterplots showing correlations between *Eubacterium hallii* and fatigue severity score, and *Cosenzaea* and fatigue severity score. The figures have been included in Figure S12 of the supplementary.

## Discussion

In this study, we identified possible link between microbiome composition and fatigue in patients with advanced cancers. Using the MD Anderson Symptom Inventory to score fatigue and 16S rRNA sequencing to characterize the intestinal microbiome of 88 patients with advanced, metastatic, unresectable cancer, we observed that *Eubacterium hallii* was negatively associated with fatigue scores whereas *Cosenzaea* was positively associated with fatigue scores.

*Eubacterium hallii*, considered a key species in trophic interactions, can highly impact metabolic balance, ultimately affecting gut microbiota and host homeostasis as well as host health^34^. In addition, *Eubacterium hallii* has been used to treat insulin resistance-related conditions, including dyslipidemia, type 1 diabetes mellitus, and Cushing syndrome, and other endocrine diseases^35^. Together, these data suggest that *Eubacterium hallii* has a role in intestinal metabolism and immune homeostasis that might impact the gut-brain axis and fatigue.

*Cosenzaea myxofaciens* (previously known as *Proteus myxofaciens*), together with the genera *Proteus*, *Providencia*, and *Morganella*, belongs to the family Enterobacteriaceae within the tribe Proteeae^36^. No data on the role of *Cosenzaea* in cancer have been reported. Under favorable conditions, *Proteus* bacteria, which inhabit the environment and are present in the intestines of humans, can cause urinary tract infections, wound infections, and meningitis (in neonates and infants). The increased abundance of *Cosenzaea myxofaciens* in high-fatigue cancer patients indicates a potential role of the bacteria in inducing inflammation.

## Limitations

This study had a relatively small sample size, which might have precluded the identification of significant differences in the diversity of the microbiome between high- and low-fatigue patients, and across different types of cancer. In addition, six patients were on antibiotics within 1–3 months of stool sample collection, which could have affected their intestinal microbiome composition. However, recent studies have suggested that antibiotic use in the preceding 30 days is not associated with alpha diversity or dysbiosis index^37^. The mean fatigue score in our cohort was lower than those previously reported for cancer patients receiving no treatment (4.52, SD = 3.3), patients receiving chemotherapy (5.2, SD = 2.81), and patients receiving bone marrow transplantations (5.29, SD = 2.55)^38^. This may be explained by the good performance status our patients were required to have to meet the eligibility criteria for enrollment on clinical trials, and this might present some selection bias. However, we were able to identify a significant difference in fatigue scores (some with severe fatigue and others with mild fatigue) even among patients with good performance status. Finally, we did not have healthy controls in this study, however, we plan to collect healthy household samples in future longitudinal studies.

## Conclusions

Our study shows that cancer patients experiencing different levels of fatigue can have distinct gut microbiome compositions. Given the importance of the microbiome in mucosal immunity and increasing recognition of the association between the gut-brain axis and fatigue and other symptoms, our findings highlight the need to further evaluate the microbiome in cancer patients with fatigue using longitudinal samples, and assess whether modulating the microbiome reduces cancer patients’ fatigue severity and improves their quality of life.

## Supplementary Information

Supplementary Information

## Data Availability

The datasets used and/or analyzed during the current study are available from the corresponding author on reasonable request according to available guidelines at the time of request.
