# Supplementary material for: Associations between the gut microbiome and fatigue in cancer patients
Source: Sci Rep. 2021 Mar 12;11:5847. doi: 10.1038/s41598-021-84783-9 (PMC7954807; doi:10.1038/s41598-021-84783-9)
Supplement: Supplementary file 1 — Supplementary Information [file 41598_2021_84783_MOESM1_ESM.pdf]

Supplementary Material for Manuscript # SREP-20-03188

Title Associations between the Gut Microbiome and Fatigue in Cancer Patients

Authors Joud Hajjar, MD; Tito Mendoza, PhD; Liangliang Zhang, PhD; Siqing Fu, MD, PhD; Sarina A. Piha-Paul, MD; David S. Hong, MD; Filip Janku, MD, PhD; Daniel D. Karp, MD; Alexej Ballhausen, MD; Jing Gong, MS; Abdulrazzak Zarifa, MD; Christine Peterson, PhD; Funda Meric-Bernstam, MD; Robert Jenq, MD, PhD, and Aung Naing, MD

1. Description of primer sequences

The V4 region of 16S rRNA gene was amplified by PCR from 100 ng of each of extracted and purified genomic DNA using 515 forward and 806 reverse primer pairs. The exact primer sequences are as follows:

- 806R GGACTACNVGGGTWTCTAAT
- 515F GTGYCAGCMGCCGCGGTAA

For more details, please refer to the web page https://earthmicrobiome.org/protocols-and-standards/16s/ and Caporaso, JG. et al. [1].

Figure S1

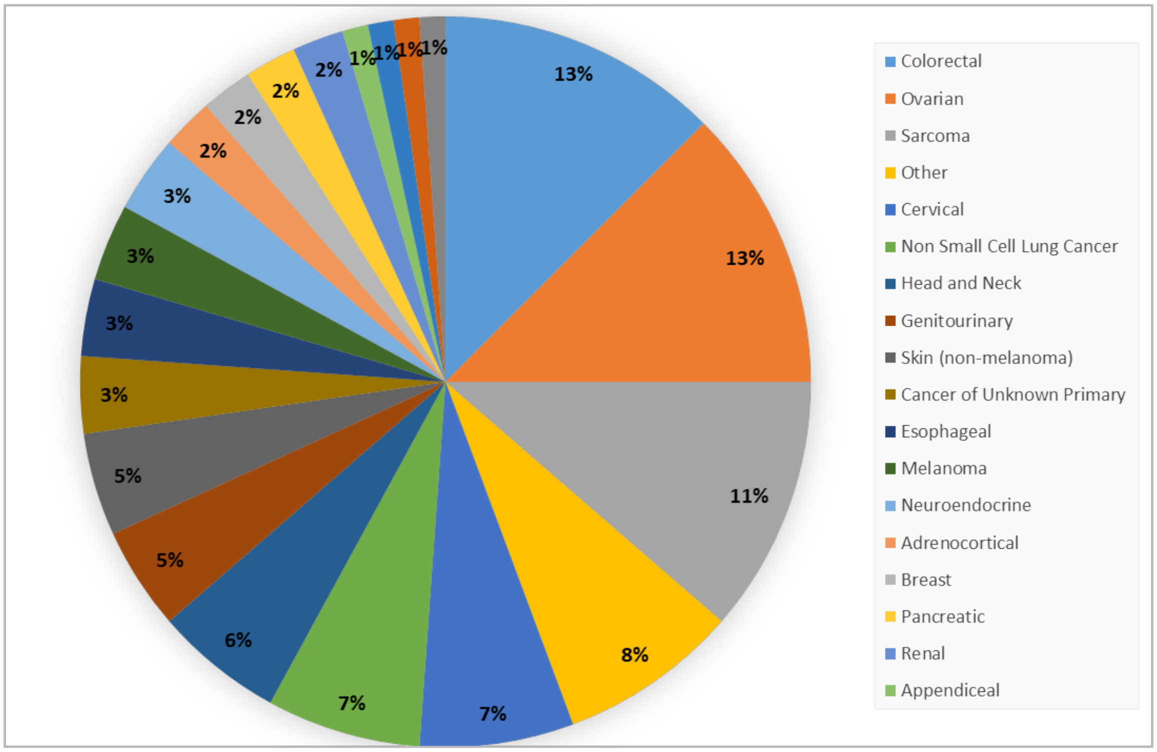

Figure S1. Cancer types among patients in the study.

Figure S2

A.

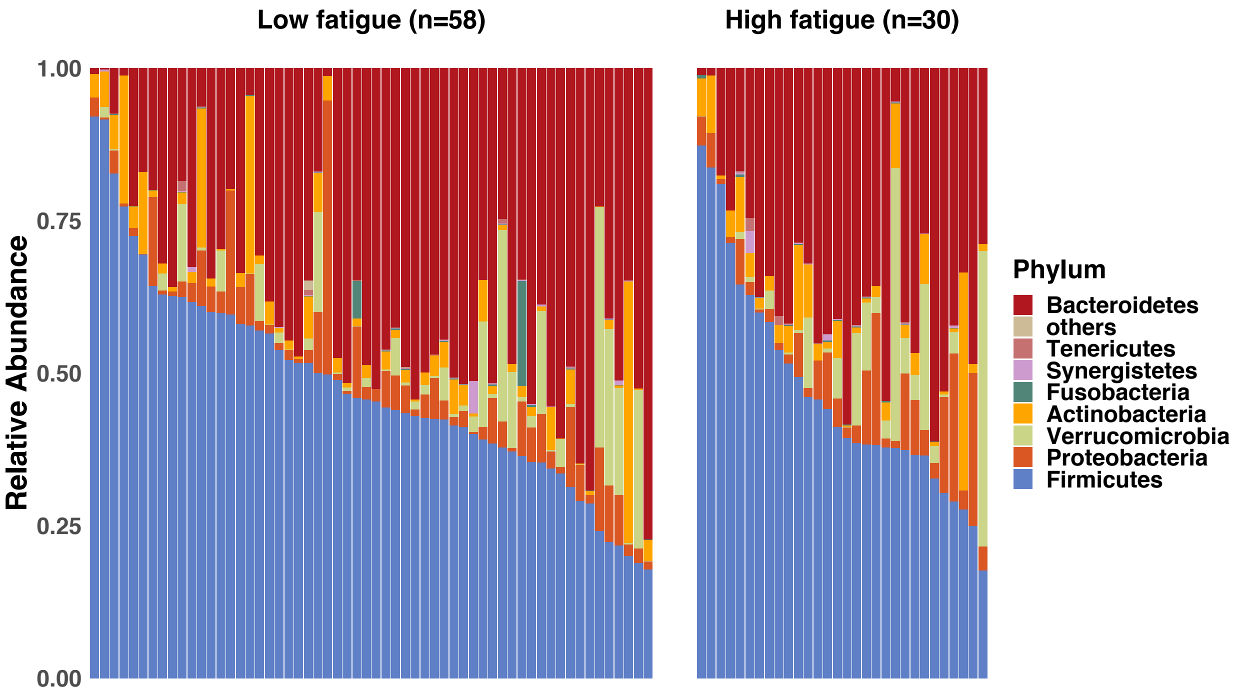

Low fatigue (n=58)
High fatigue (n=30)
Relative Abundance
1.00
0.75
0.50
0.25
0.00
Phylum
Bacteroidetes
others
Tenericutes
Synergistetes
Fusobacteria
Actinobacteria
Verrucomicrobia
Proteobacteria
Firmicutes

B.

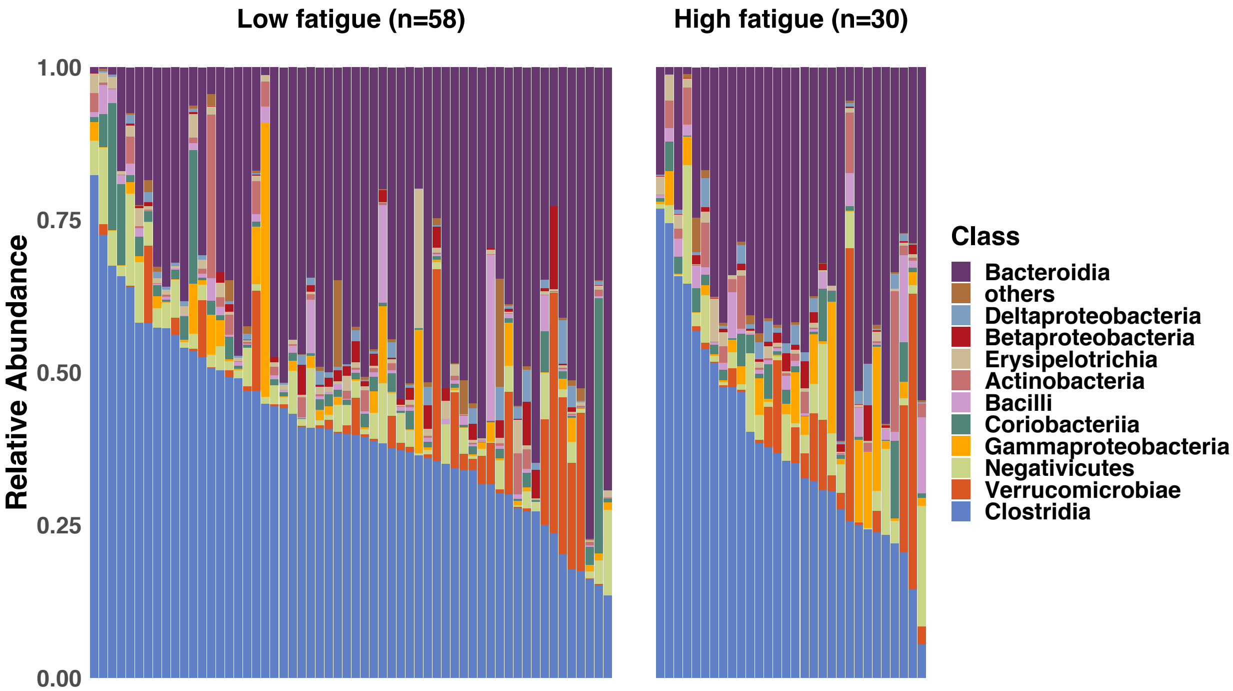

Low fatigue (n=58)
High fatigue (n=30)
Relative Abundance
1.00
0.75
0.50
0.25
0.00
Class
Bacteroidia
others
Deltaproteobacteria
Betaproteobacteria
Erysipelotrichia
Actinobacteria
Bacilli
Coriobacteriia
Gammaproteobacteria
Negativicutes
Verrucomicrobiae
Clostridia

C.

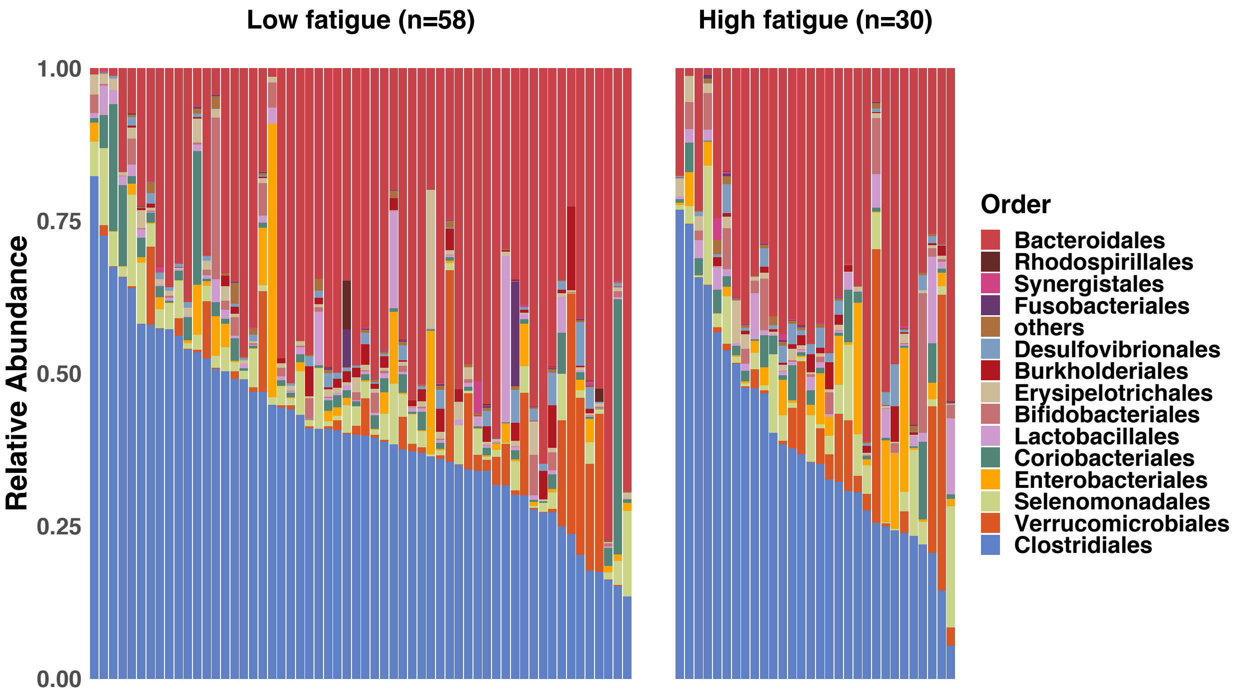
Low fatigue (n=58)
High fatigue (n=30)
Relative Abundance
1.00
0.75
0.50
0.25
0.00
Order
Bacteroidales
Rhodospirillales
Synergistales
Fusobacteriales
others
Desulfovibrionales
Burkholderiales
Erysipelotrichales
Bifidobacteriales
Lactobacillales
Coriobacteriales
Enterobacteriales
Selenomonadales
Verrucomicrobiales
Clostridiales

D.

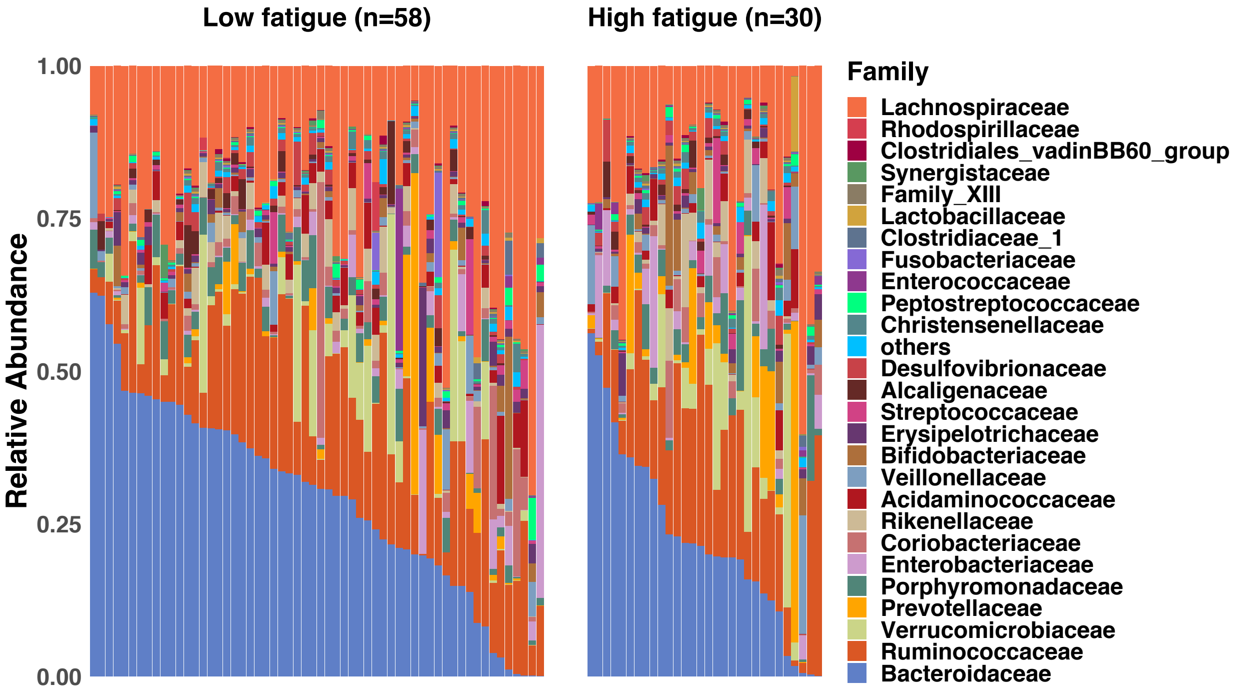
Low fatigue (n=58)
High fatigue (n=30)
Relative Abundance
1.00
0.75
0.50
0.25
0.00
Family
Lachnospiraceae
Rhodospirillaceae
Clostridiales_vadinBB60_group
Synergistaceae
Family_XIII
Lactobacillaceae
Clostridiaceae_1
Fusobacteriaceae
Enterococcaceae
Peptostreptococcaceae
Christensenellaceae
others
Desulfovibrionaceae
Alcaligenaceae
Streptococcaceae
Erysipelotrichaceae
Bifidobacteriaceae
Veillonellaceae
Acidaminococcaceae
Rikenellaceae
Coriobacteriaceae
Enterobacteriaceae
Porphyromonadaceae
Prevotellaceae
Verrucomicrobiaceae
Ruminococcaceae
Bacteroidaceae

E.

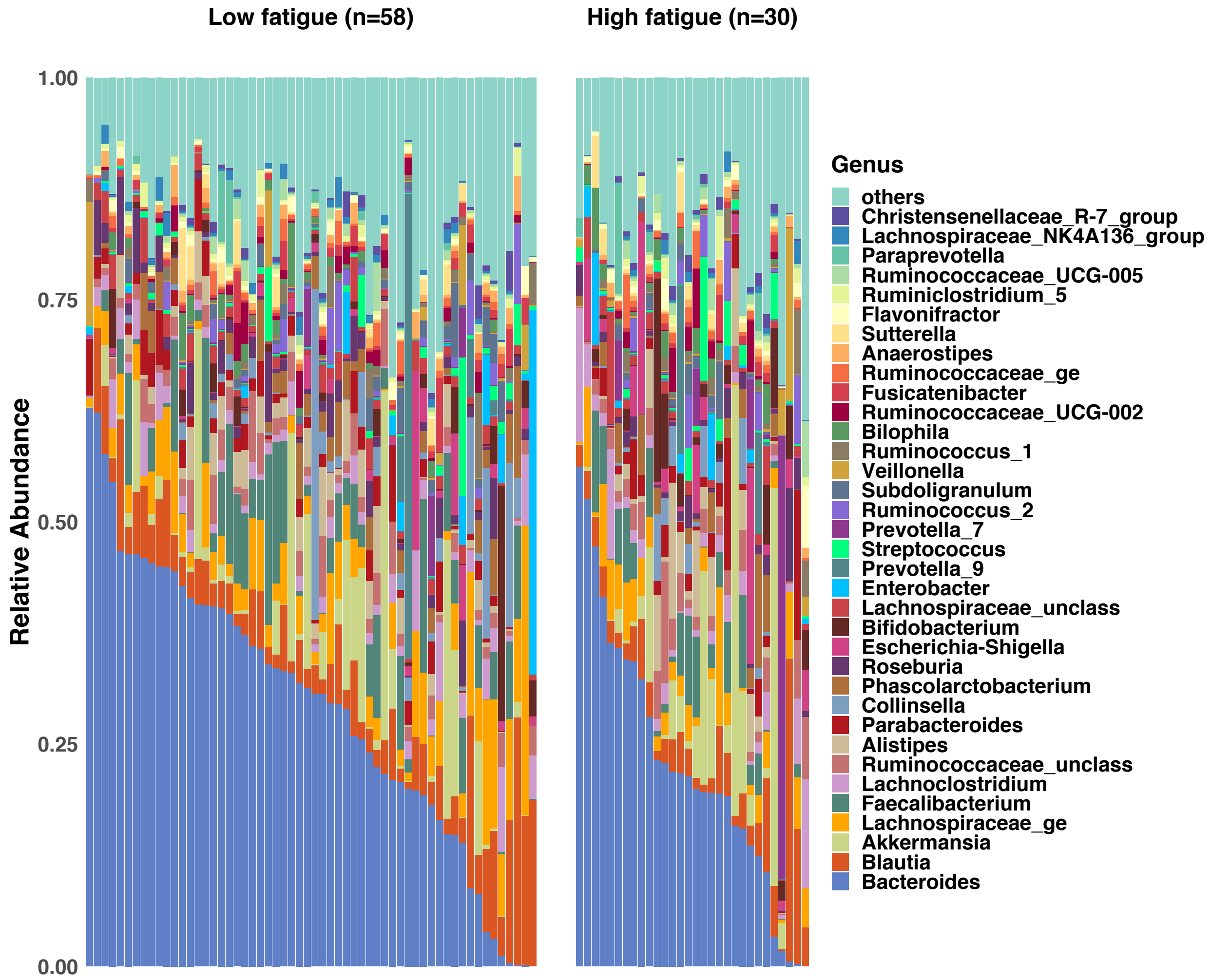

Figure S2. Taxonomic Composition Based on Influencing Factors.

(A-E) Bar plots displaying taxonomic compositions in low- and high- fatigue groups at phylum level (A), class level (B), order level (C), family level (D), and genus level (E). Relative abundance is plotted for each sample (0%-100%)

Figure S3

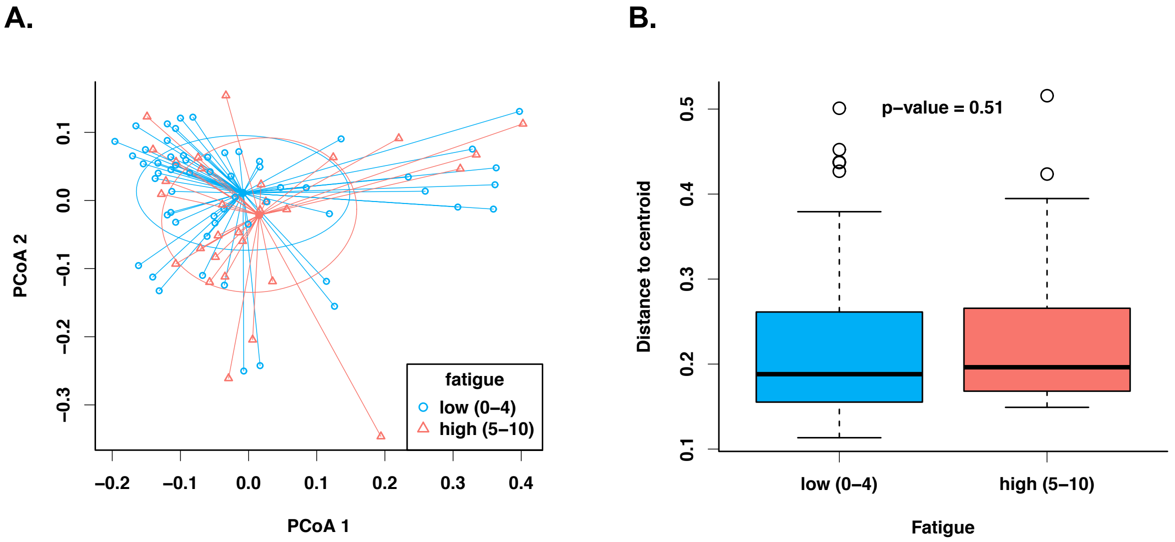

Figure S3. Comparisons of the dispersion of weighted-UniFrac distances between two groups.

A. Principle component analysis (PCoA) plot with individual distances to the centroid shows the inter-individual variations. Each blue dot represents the PCoA score of one patient in the low-fatigue group; Each red triangle represents the PCoA score of one patient in the high-fatigue group.

B. Boxplot of individual-centroid distances. The p-value is given by Tukey test. The box with blue color describes the distance distribution in the low fatigue group; The box with red color describes the distance distribution in the high fatigue group.

Figure S4

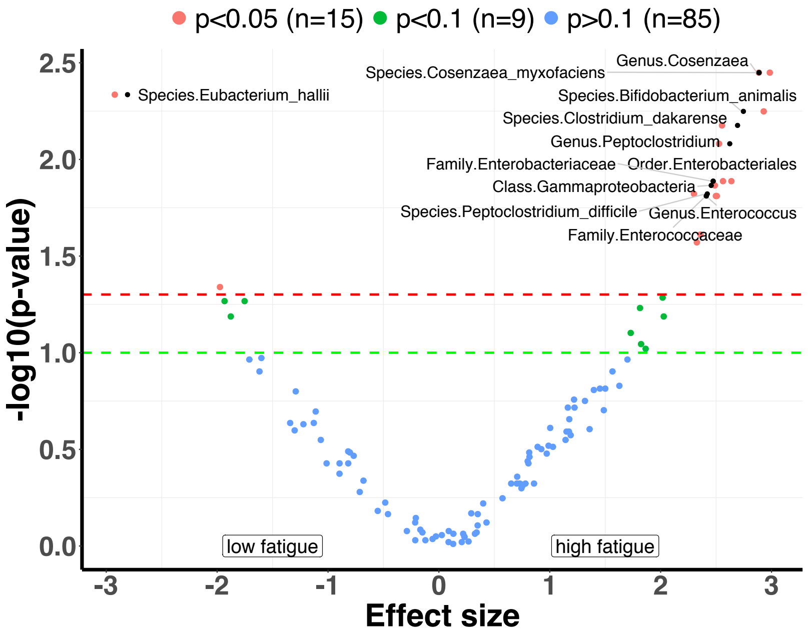

Figure S4. Volcano plot showing that, of 109 taxonomic features, 15 were significantly differential between low- and high-fatigue groups ($p<0.05$; Mann-Whitney U test). Progressive permutation analysis revealed a subgroup of 12 features (labeled) to be robust findings. The blue dot represents the features whose p-values are greater than 0.1; the green dot represents the features whose p-values are less than 0.1 and greater than 0.05; the red dot represents the features whose p-value are less than 0.05. A positive effect size indicates that the microbial feature is more abundant in high-fatigue group.

Figure S5

A.

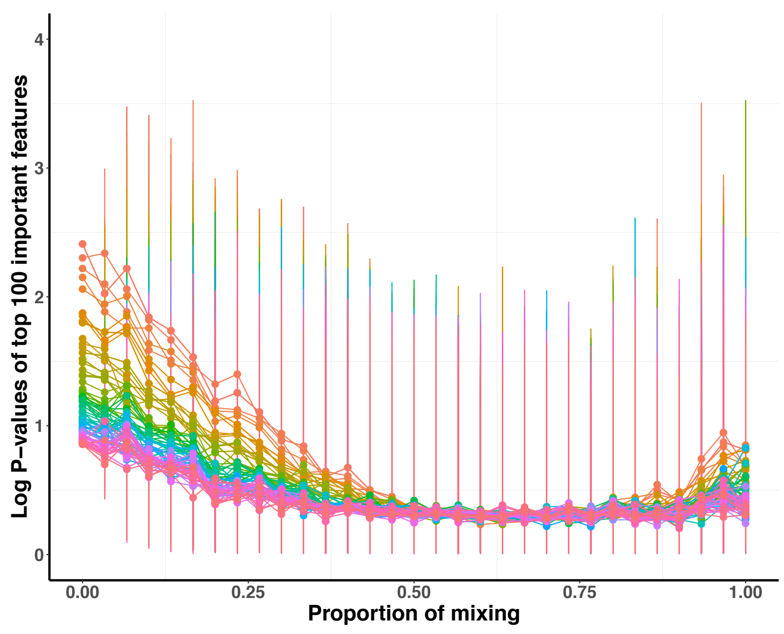

Log P−values of top 100 important features
0
1
2
3
4
0.00
0.25
0.50
0.75
1.00
Proportion of mixing

B.

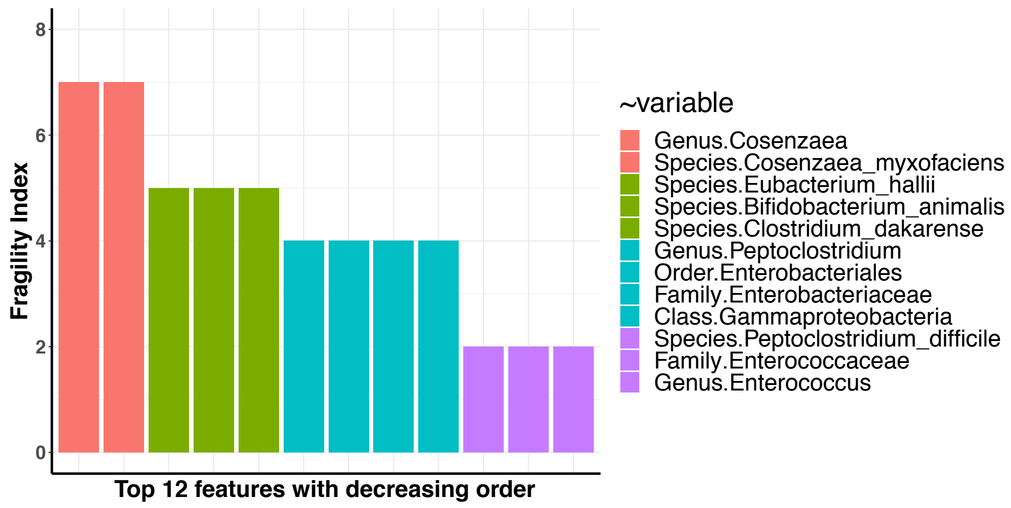

Fragility Index
0
2
4
6
8
Top 12 features with decreasing order
~variable
Genus.Cosenzaea
Species.Cosenzaea_myxofaciens
Species.Eubacterium_hallii
Species.Bifidobacterium_animalis
Species.Clostridium_dakarense
Genus.Peptoclostridium
Order.Enterobacteriales
Family.Enterobacteriaceae
Class.Gammaproteobacteria
Species.Peptoclostridium_difficile
Family.Enterococcaceae
Genus.Enterococcus

C.

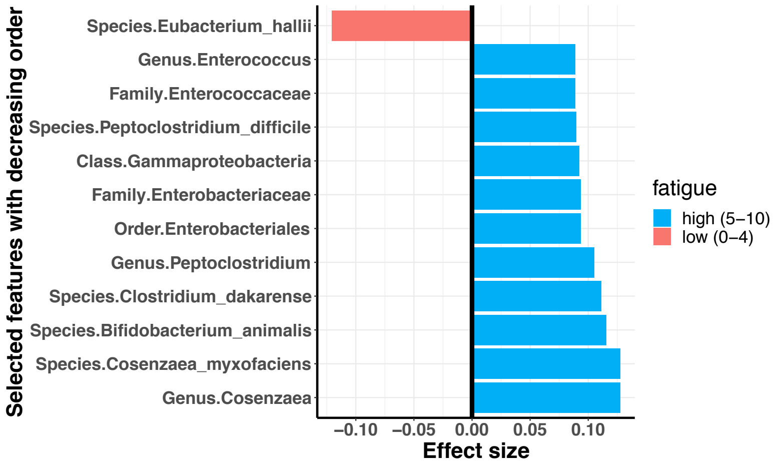

Figure S5. Progressive permutation analysis on concluding the robustness of the findings

A. Trace plot displaying the -Log10 P-values of the top 100 features with incremental proportion of mixing samples

B. Bar plot depicting the fragility indices of the top 20 features with a decreasing order

C. Bar plot of effect sizes of the selected features. The blue bar denotes positive effect sizes, while the red bar denotes negative effect sizes.

Figure S6

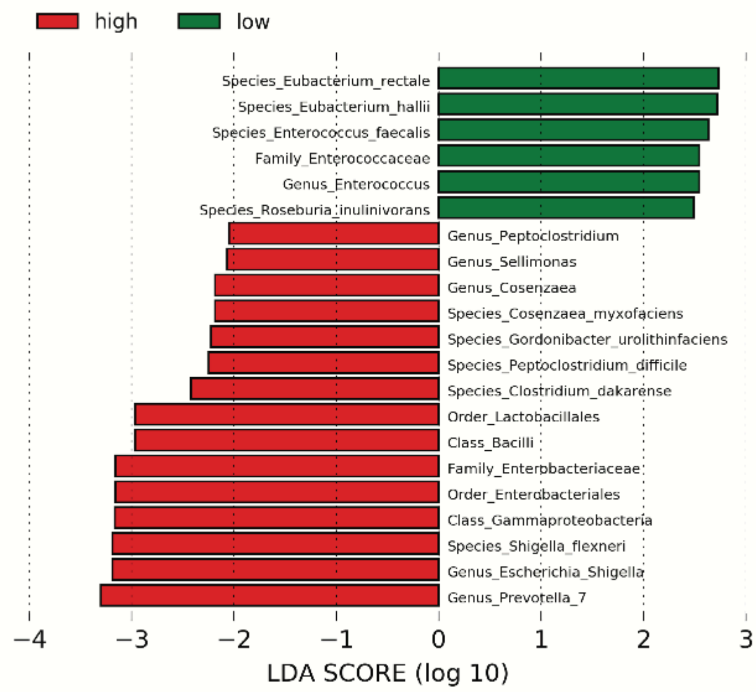

Figure S6. Bar plot of effect sizes of the features by LEfSe. The green bar denotes that the microbial feature is more abundant in the low fatigue group, while the red bar denotes that the microbial feature is more abundant in the high fatigue group.

Figure S7

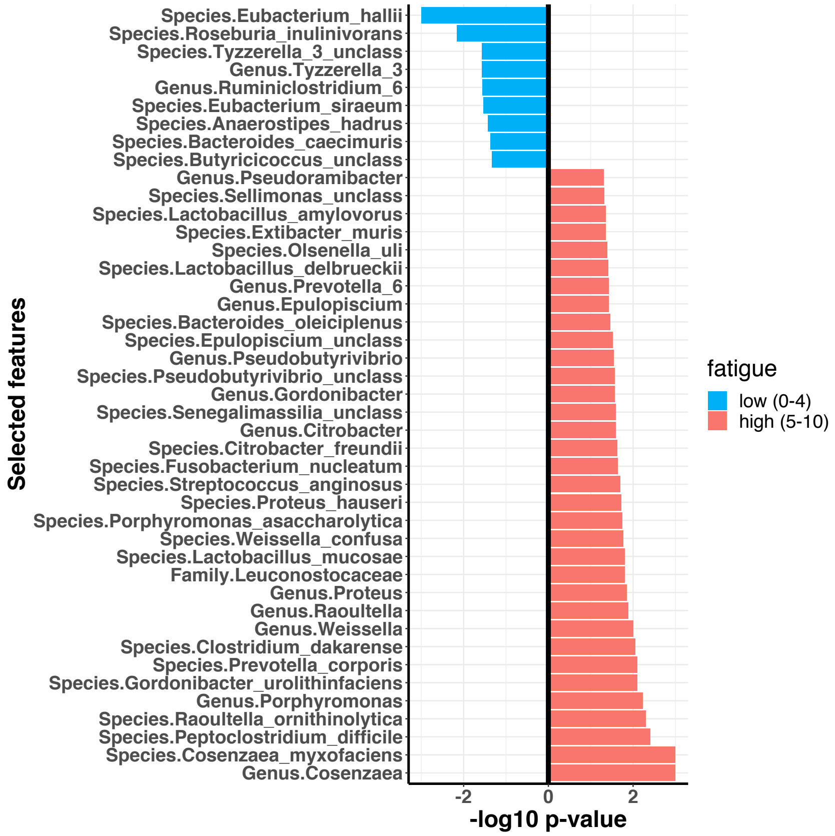

Figure S7. Bar plot of effect sizes of the features selected by labdav. The blue bar denotes that the microbial feature is the best indicator for the low fatigue group, while the red bar denotes that the microbial feature is the best indicator for the high fatigue group.

Figure S8

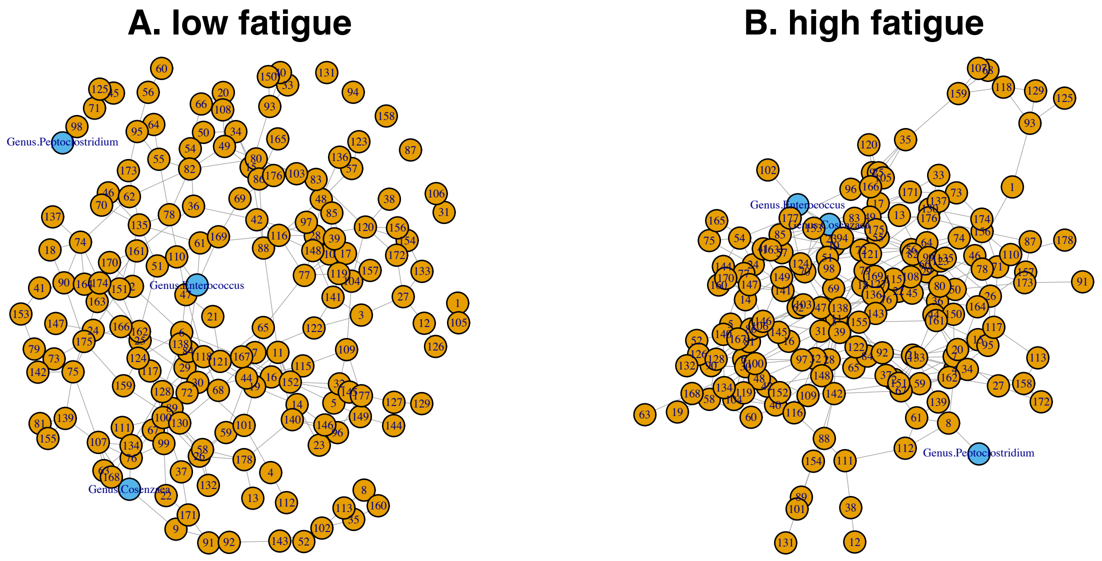

Figure S8. A network analysis using co-occurring genus using SpiecEasi. The blue vertex denotes that the microbial feature is identified as significant hits by differential tests, while the yellow vertex denotes all the other microbial features.

Figure S9

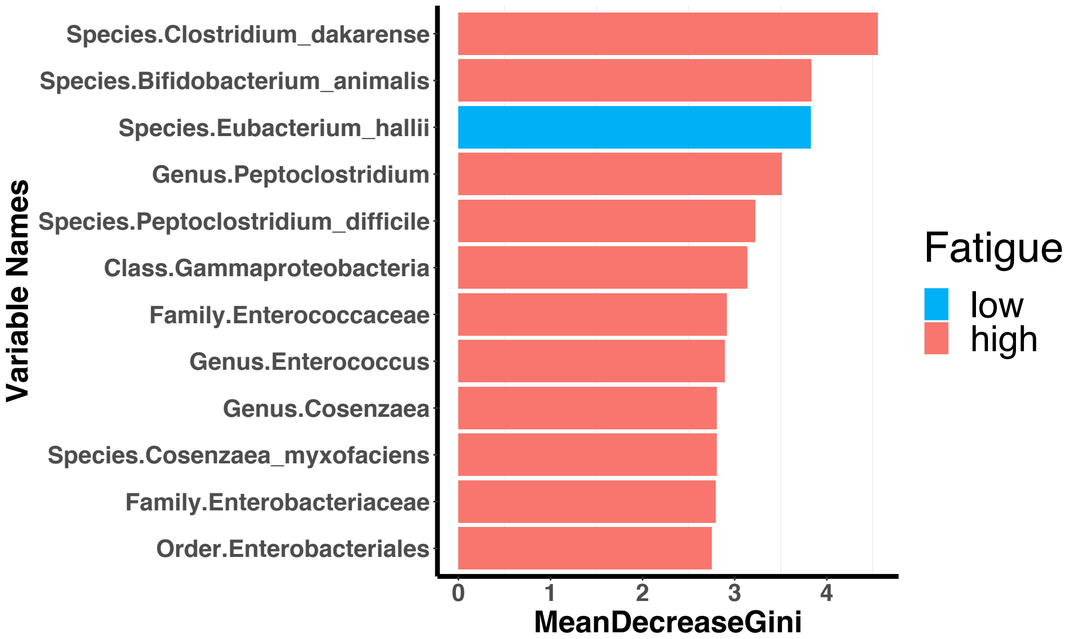

Figure S9. Bar plot of Gini index of the 12 features based on Random Forest model. The blue bar denotes that the microbial feature is more abundant in the low fatigue group, while the red bar denotes that the microbial feature is more abundant in the high fatigue group.

Figure S10

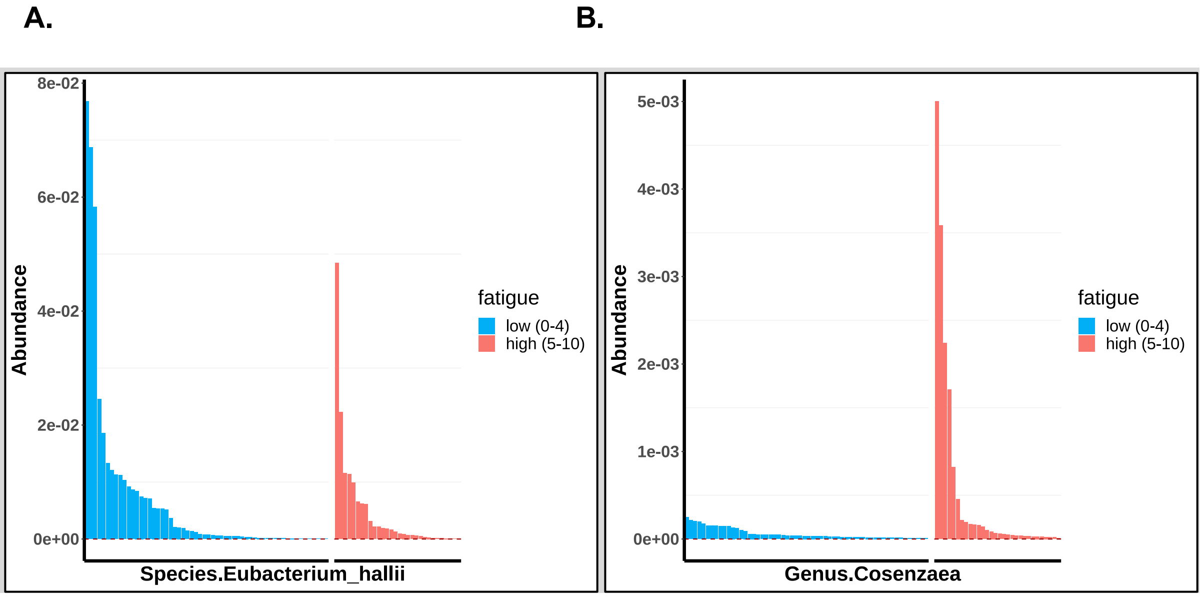

Figure S10. Bar plots describing the distribution of microbiome composition in two groups.

A. Bar plots showing differences in Eubacterium hallii abundance by specimen between the high- and low-fatigue groups.

B. Bar plots showing differences in Cosenzaea abundance by specimen between the high- and low-fatigue groups.

Figure S11

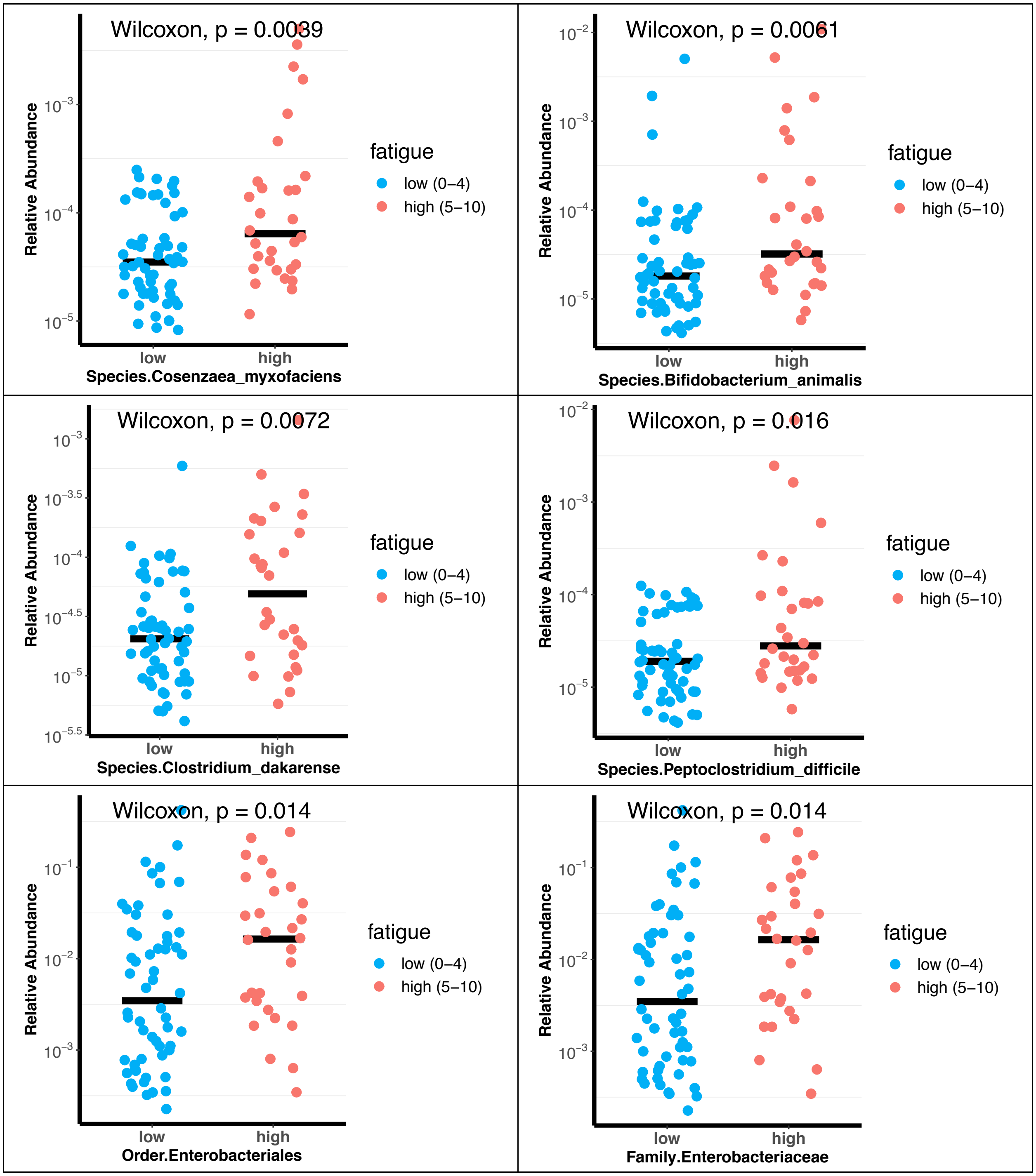

Wilcoxon, p = 0.0039
Relative Abundance
10^{-3}
10^{-4}
10^{-5}
low
high
Species.Cosenzaea_myxofaciens
fatigue
low (0–4)
high (5–10)
Wilcoxon, p = 0.0061
Relative Abundance
10^{-2}
10^{-3}
10^{-4}
10^{-5}
low
high
Species.Bifidobacterium_animalis
fatigue
low (0–4)
high (5–10)
Wilcoxon, p = 0.0072
Relative Abundance
10^{-3}
$10^{-3.5}$
10^{-4}
$10^{-4.5}$
10^{-5}
$10^{-5.5}$
low
high
Species.Clostridium_dakarense
fatigue
low (0–4)
high (5–10)
Wilcoxon, p = 0.016
Relative Abundance
10^{-2}
10^{-3}
10^{-4}
10^{-5}
low
high
Species.Peptoclostridium_difficile
fatigue
low (0–4)
high (5–10)
Wilcoxon, p = 0.014
Relative Abundance
10^{-1}
10^{-2}
10^{-3}
low
high
Order.Enterobacteriales
fatigue
low (0–4)
high (5–10)
Wilcoxon, p = 0.014
Relative Abundance
10^{-1}
10^{-2}
10^{-3}
low
high
Family.Enterobacteriaceae
fatigue
low (0–4)
high (5–10)

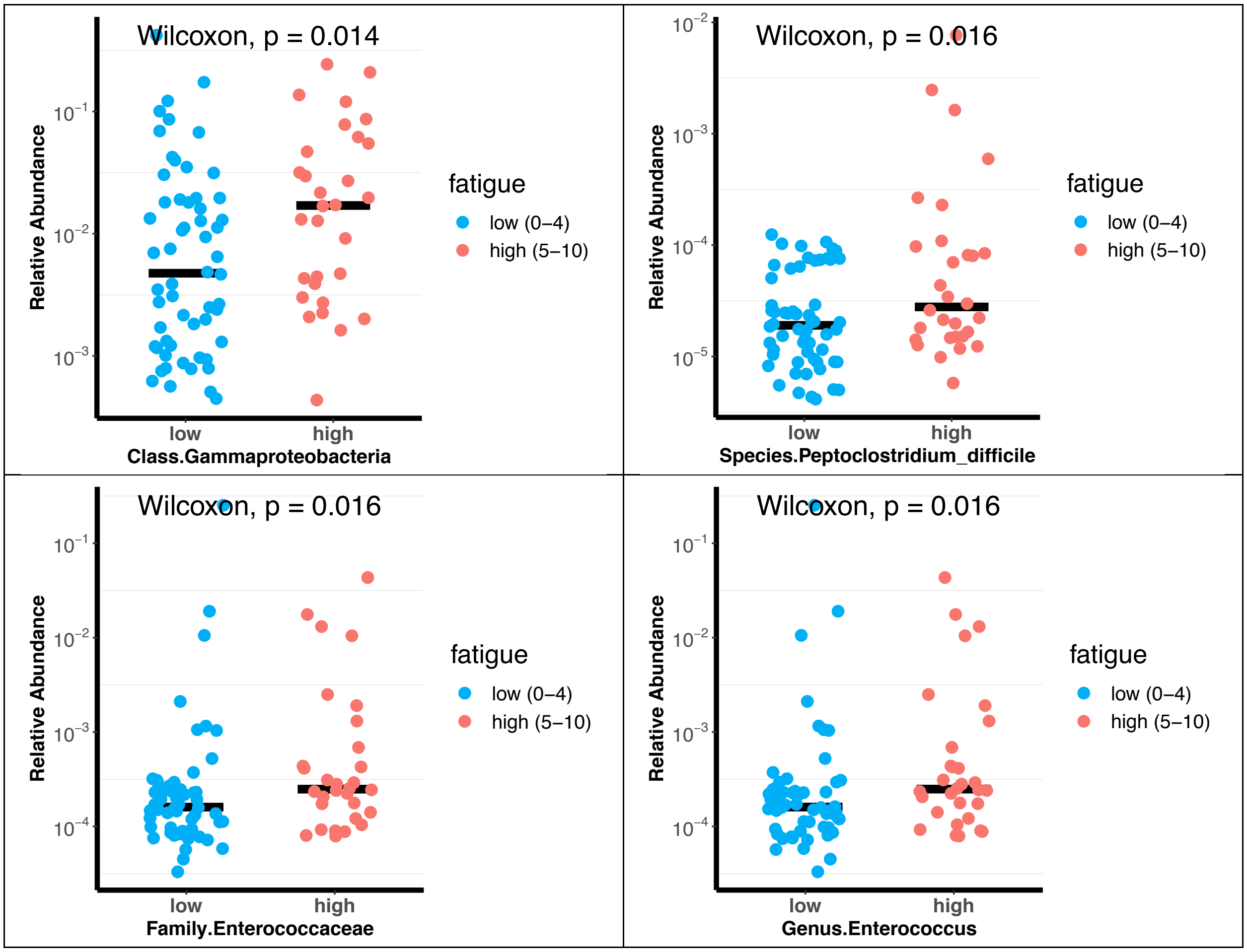

Figure S11. Dot plots showing differences in taxonomic features between the high- and low-fatigue groups. Each blue dot represents one patient in the low-fatigue group; each red dot represents one patient in the high-fatigue group. The black bar denotes the median. In all figures, we performed log10 transformation of the data so that the data points are evenly distributed. The numbers on the y-axis denotes the original quantity of relative abundance, which are just the scientific format of the abundance proportions.

Figure S12

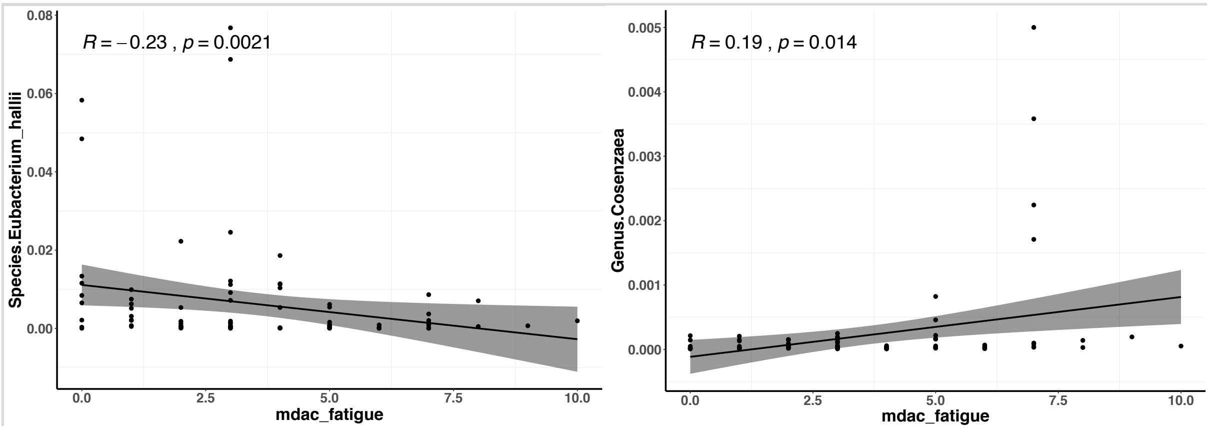

Figure S12. Scatter plots showing the correlation between taxonomic features and the fatigue severity score. The line crossing the points is the regression line. The shaded area denotes the 95% confidence band. The correlation coefficient and p-value shown on the top are obtained from Kendall correlation test.

A. Scatter plot showing correlations between Eubacterium hallii and fatigue severity score.

B. Scatter plot showing correlations between Cosenzaea and fatigue severity score.

Reference:

1. Caporaso JG, Lauber CL, Walters WA, Berg-Lyons D, Huntley J, Fierer N, Owens SM, Betley J, Fraser L, Bauer M, Gormley N. Ultra-high-throughput microbial community analysis on the Illumina HiSeq and MiSeq platforms. The ISME journal. 2012 Aug;6(8):1621-4.
